# Supplementary figures and images for: Integrative omics reveals subtle molecular perturbations following ischemic conditioning in a porcine kidney transplant model
Source: Clin Proteomics. 2022 Feb 14;19:6. doi: 10.1186/s12014-022-09343-3 (PMC8903695; doi:10.1186/s12014-022-09343-3)

## Slide 1
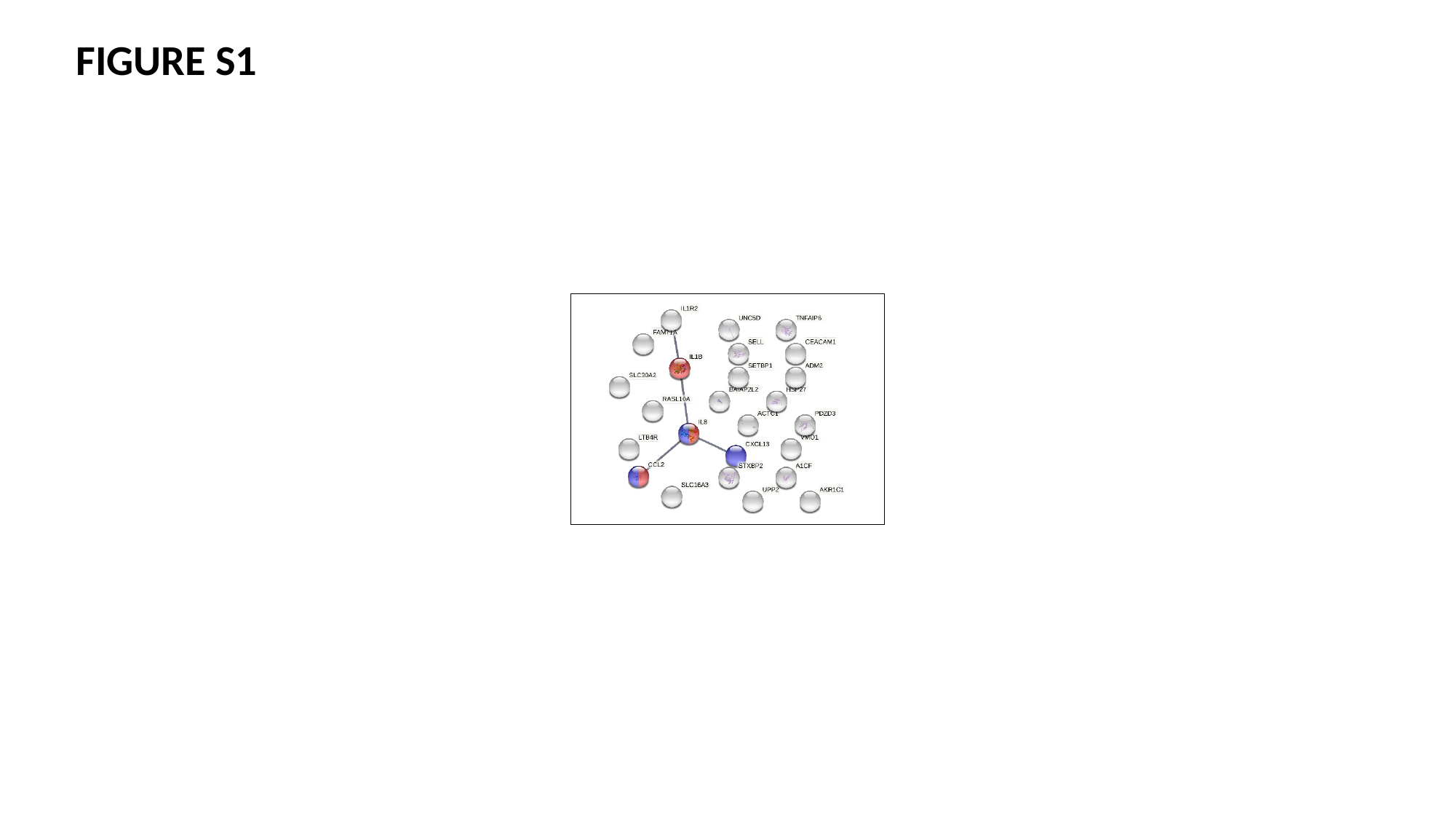

FIGURE S1

## Slide 2
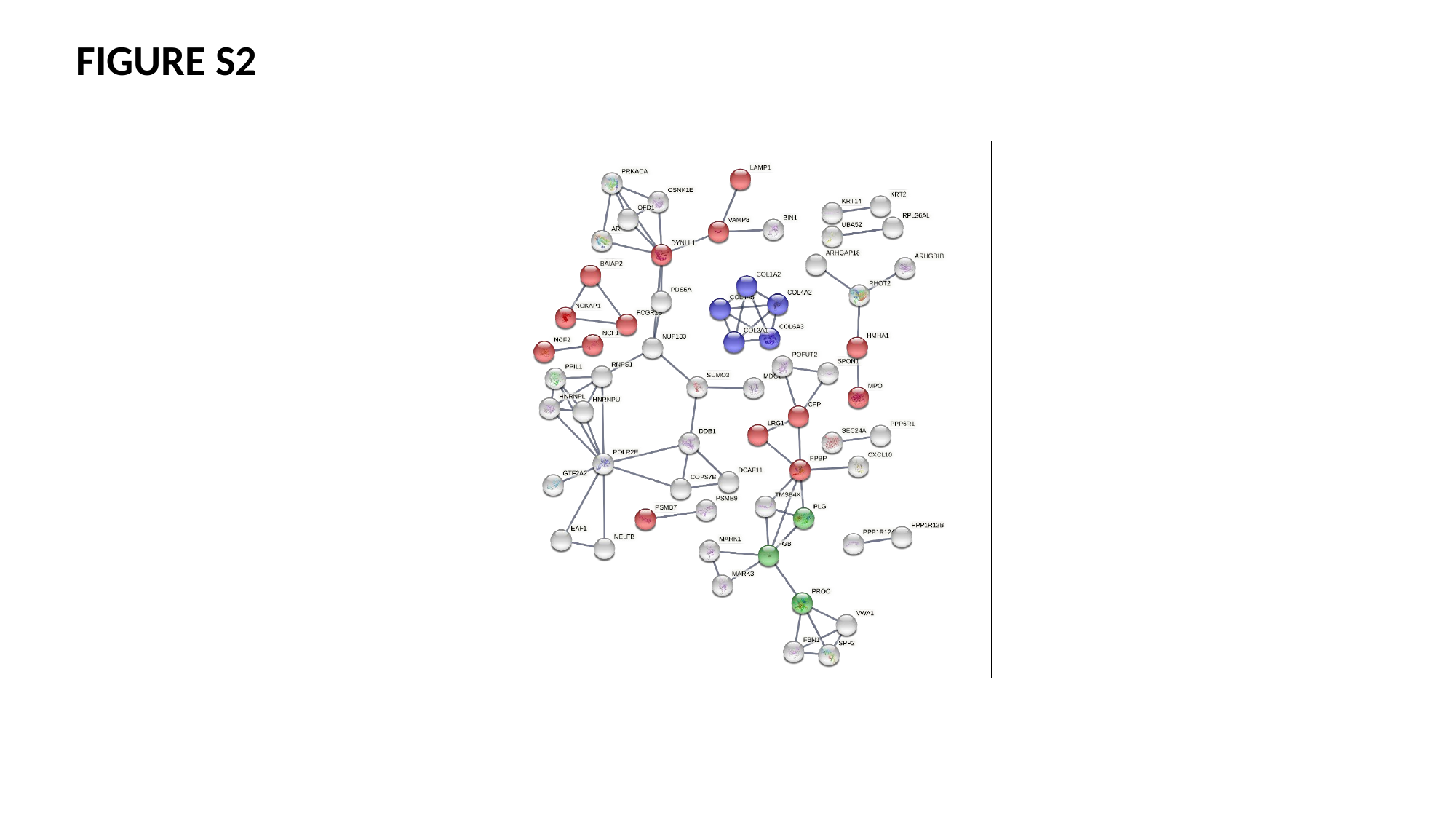

FIGURE S2

## Slide 3
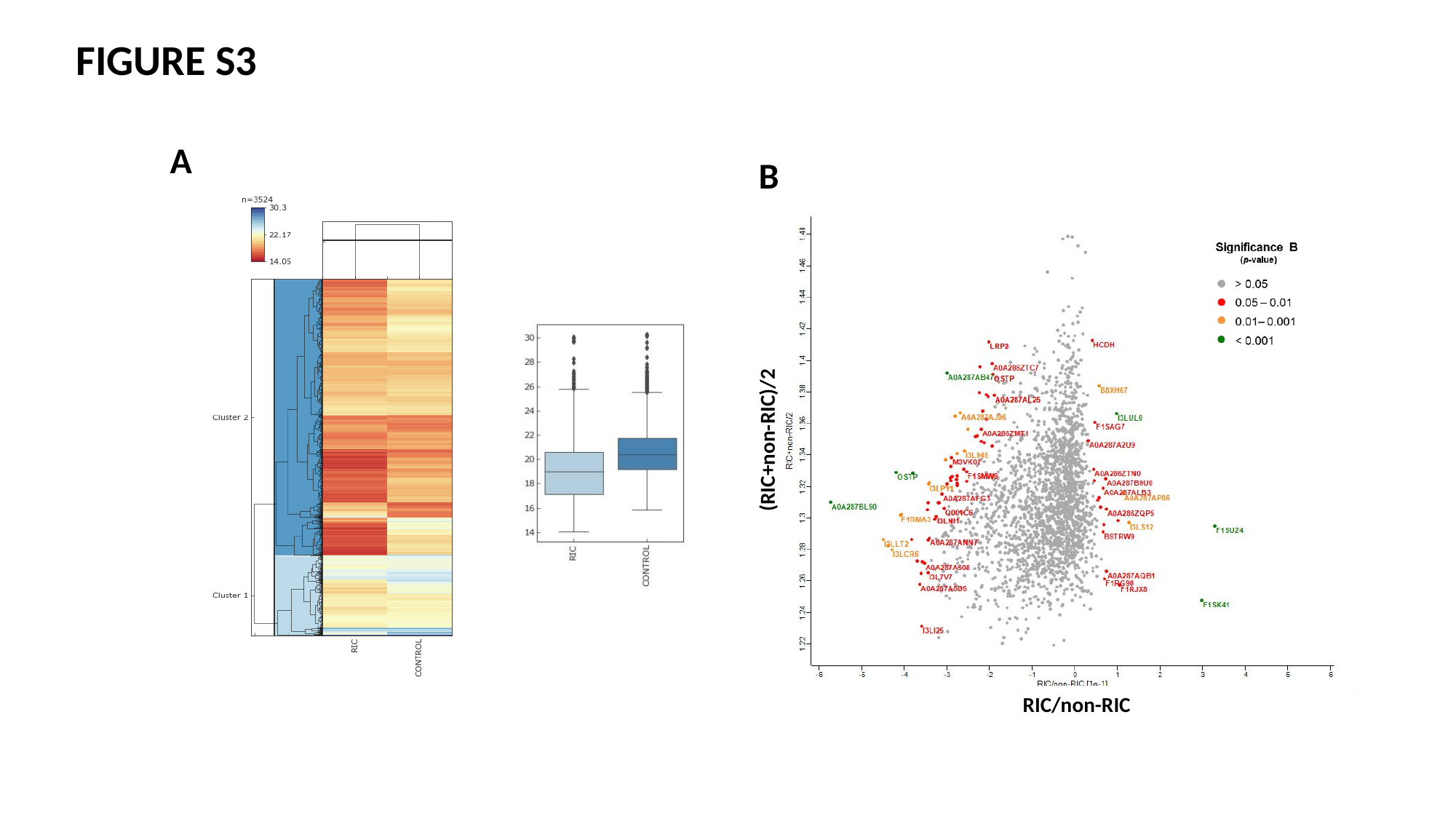

FIGURE S3
A
B
(RIC+non-RIC)/2
RIC/non-RIC

Supplement: Supplementary file 1 — Additional file 1: Figure S1. Transcriptomic pathway enrichment analysis reveals subtle alteration of tissue inflammation by RIC. All transcripts (n = 33) which were found to be dysregulated in RIC versus non-RIC controls were searched against the Sus scrofa database in STRING. Only interactions of the highest confidence (scores > 0.90) were included in the analysis. Genes associated with immune regulation are highlighted, with those linked to interleukin biology coloured red, while cytokines are coloured purple. Figure S2. Proteomic pathway enrichment analysis uncovers RIC induced tissue leakage and altered inflammation. Proteins (n = 252) which were found to have the greatest dysregulation in RIC versus non-RIC controls were searched against the Sus scrofa database in STRING. Only interactions of the highest confidence (scores > 0.90) were included in the analysis. Proteins/genes of interest are highlighted, with proteins associated with muscle and ECM coloured in blue, blood coagulation in green, and factors associated with innate immunity coloured red. Figure S3. Kidney tissue phosphoproteomics unaffected by RIC. A) Abundance plots indicate no significant difference in the phosphoproteome between RIC and control groups. B) A Christmas tree plot, with Significance B thresholds colour coded. Minor changes were observed between groups. [file 12014_2022_9343_MOESM1_ESM.pptx]
